# Cost-effectiveness analysis of a postoperative clinical care pathway in head and neck surgery with microvascular reconstruction

**DOI:** 10.1186/1916-0216-42-59

**Published:** 2013-12-19

**Authors:** Jonathan F Dautremont, Luke R Rudmik, Justin Yeung, Tiffany Asante, Steve C Nakoneshny, Monica Hoy, Amanda Lui, Shamir P Chandarana, Thomas W Matthews, Christiaan Schrag, Joseph C Dort

**Affiliations:** 1 Division of Otolaryngology-Head and Neck Surgery, Department of Surgery, University of Calgary, Calgary, AB, Canada; 2 Division of Plastic Surgery, Department of Surgery, University of Calgary, Calgary, AB, Canada; 3 Bachelor of Health Sciences Program, Faculty of Medicine, University of Calgary, Calgary, AB, Canada; 4 Ohlson Research Initiative, Southern Alberta Cancer Research Institute, Faculty of Medicine, University of Calgary, Calgary, AB, Canada; 5 HRIC 2A02, 3280 Hospital Dr, Calgary T2N 4Z6, NW, Canada

## Abstract

**Background:**

The objective of this study is to evaluate the cost-effectiveness of a postoperative clinical care pathway for patients undergoing major head and neck oncologic surgery with microvascular reconstruction.

**Methods:**

This is a comparative trial of a prospective treatment group managed on a postoperative clinical care pathway and a historical group managed prior to pathway implementation. Effectiveness outcomes evaluated were total hospital days, return to OR, readmission to ICU and rate of pulmonary complications. Costing perspective was from the government payer.

**Results:**

118 patients were included in the study. All outcomes demonstrated that the postoperative pathway group was both more effective and less costly, and is therefore a dominant clinical intervention. The overall mean pre- and post-pathway costs are $22,733 and $16,564 per patient, respectively. The incremental cost reduction associated with the postoperative pathway was $6,169 per patient.

**Conclusion:**

Implementing the postoperative clinical care pathway in patients undergoing head and neck oncologic surgery with reconstruction resulted in improved clinical outcomes and reduced costs.

## Background

The treatment of head and neck squamous cell carcinoma (HNSCC) is among the most expensive of all solid malignant tumors and places a significant burden on the healthcare system [1]. The majority of the treatment expenditure is consumed by hospitalization costs, particularly in the postoperative period [1]. Postoperative care in these patients is complex, with lengthy hospitalization and recovery periods. Coordination with several health care specialties, including nursing, physiotherapy, and respiratory therapists is required. Due to the growing concern about the fiscal sustainability of health care systems around the globe, identifying clinical interventions to improve the cost-effectiveness of managing HNSCC is important [2].

A clinical care pathway is an evidence-based algorithm designed by compiling input from multiple health care disciplines. The pathway defines the health care milestones that are required to provide a patient, who has a specific diagnosis, with high quality care [3]. The pathway determines the timing and co-ordination of all essential interventions provided by multiple health care disciplines to minimize delays, omissions and duplications and therefore maximize effectiveness and efficiency [3,4].

The objective of this study was to evaluate the cost-effectiveness of a postoperative clinical care pathway for patients undergoing major head and neck oncologic surgery with microvascular reconstruction. We hypothesize that implementation of a postoperative clinical care pathway will be cost-effective.

## Methods

The study was comprised of two distinct patient populations; a historical (pre-pathway) control cohort, and the treatment cohort of patients managed following the implementation of the pathway. The medical charts of the pre-pathway group, consisting of 62 patients treated between January 2005 and December 2009, were reviewed. The prospective, care pathway group consisted of 56 patients who underwent head and neck oncologic resection with microvascular reconstruction between January 2011 and May 2012. Clinical outcomes data was prospectively collected on this group. Patients treated between January 2010 and December 2010 were not included, as the care pathway was being designed, implemented and revised during this time period. Enrollment of patients for the treatment group began once the care pathway was fully implemented in its final form, in order to achieve the most accurate assessment of the pathway effect. E.

The inclusion criteria were all patients having undergone resection for HNSCC, with reconstruction of the defect performed using microvascular surgery (ie free flap reconstruction). There were no exclusion criteria. Patients were not excluded from the trial if their care deviated from the guidelines laid out by the care pathway. This allows for a pragmatic appraisal of the pathway’s efficacy.

The finalized version of the postoperative care pathway is shown in Additional file 1. The pathway itself was conceived and developed by fellowship-trained head and neck oncology surgeons at the University of Calgary, with input from other experienced health care professionals.

A design and trial period to allow staff, residents and physicians to become aware and familiar with the pathway occurred from January 2010 to December 2010. To minimize potential study bias, the majority of nursing and allied health staff were unaware of the study, with the exception of the head and neck unit nursing manager, and attending and resident physicians directly involved with the study. Patients treated during this trial period were not included in the study.

All patients were either admitted to hospital the day of surgery, or were admitted prior to surgery due to complications arising from the malignancy (i.e. airway obstruction). Resection in both the control and the study group were performed by a head and neck oncology fellowship trained otolaryngologist, while reconstruction was performed either by a plastic surgeon trained in microvascular surgery, an otolaryngologist with microvascular training, or both a plastic surgeon and an otolaryngologist working in conjunction. All resections and reconstructions were performed on the same day. Following the surgery, patients in both groups were taken immediately to the intensive care unit under sedation via morphine and midazolam infusion. Patients were discharged to the head and neck surgery unit once they had been successfully weaned off ventilation support and sedation. Postoperative day 1 of the care pathway was therefore initiated in the ICU.

Data was collected on patient demographics, tumor site, disease staging, and reconstruction performed. Outcomes data collected included length of stay, time to decannulation, flap failure requiring return to OR, ICU readmission, and rate of pneumonia. Pneumonia was selected as a key complication to be investigated, as it is one of the more common complications seen in head and neck surgery, and is attributed to significant morbidity, increased length of stay and mortality [5-9]. Pneumonia was defined by either typical radiographic changes or positive sputum culture, plus two of: fever, increased oxygen requirement, or elevated white blood cell count >11x10 [9] cells/L.

All statistical analyses were performed using Stata 12.1 (StataCorp LP, College Station, TX). Two-sided tests were used to analyze all clinical outcomes data. Chi-squared, Fisher’s exact, and student’s t-tests were used to analyze the various clinical outcomes. This study received ethical approval from The University of Calgary Conjoint Health Research Ethics Board. Written informed consent was obtained from all patients for the publication of this report.

### Cost collection and analysis

Costing perspective was the government payer. Intraoperative and postoperative costs were defined using the time-driven activity based costing approach [10,11]. Resource quantification was performed by retrospective review of the electronic medical records of each patient. Resource unit prices were defined by reviewing the Alberta Health Services (AHS) purchasing database, and by contacting the AHS pharmacy, Calgary Lab Services, and the AHS diagnostic imaging department. Practical capacity cost rates were defined by contacting each of the respective staffing unions and the provincial physician fee schedule. Operative and postoperative care times were defined by retrospective review of the nursing records.

## Results

A total of 118 subjects met the inclusion criteria and were included in the study. As shown in Table 1, patient demographics and disease characteristics were comparable in both the control and study group. There were no differences in tumor site, staging or type of reconstruction. The majority of procedures performed were oral cavity excisions with neck dissection, and the most common reconstructive technique used was microvascular reconstruction with radial forearm free flap.

**Table 1:** Frequency distribution of characteristics of patients undergoing head and neck cancer resection with microvascular reconstruction

	**Control *(n = 62)***	**Pathway *(n = 56)***	**p value**
**Age (mean)**	62.6	62.6	ns
	**% (n)**	**% (n)**	
**Gender**			ns
**Male**	50.0 (31)	33.9	
**Female**	50.0 (31)	66.1 (37)	
**Primary tumor site**			ns
**Oral cavity**	83.9 (52)	76.8 (43)	
**Other**	16.1 (10)	23.2 (13)	
**Staging of disease**			ns
**Stage I/II**	22.6 (14)	32.1 (18)	
**Stage III/IV**	75.8 (47)	66.1 (37)	
**Resection of mandible**	35.5 (22)	39.3 (22)	ns
**Reconstructive type**			ns
**Radial forearm**	56.5 (35)	62.5 (35)	
**Fibula**	19.4 (12)	12.5 (7)	
**Anterolateral thigh**	8.1 (5)	16.1 (9)	
**Other free flap**	16.1 (10)	8.9 (5)	
**ASA**			ns
**1**	6.5 (4)	5.4 (3)	
**2**	40.3 (25)	44.6 (25)	
**3**	48.4 (30)	42.8 (24)	
**4**	3.2 (2)	0 (0)	
**No ASA recorded**	1.6 (1)	7.1 (4)	
**Alcohol history**	59.7 (37)	73.2 (41)	ns
**Smokers**	58.1 (36)	62.5 (35)	ns
**COPD**	22.6 (14)	23.2 (13)	ns
**Diabetes Mellitus**	4.8 (3)	10.7 (6)	ns

Patients in the care pathway group had a significantly shorter inpatient length of stay (LOS) in the postoperative period, at a mean of 14.1 days compared to 21.3 days in the pre-pathway group (p < 0.001). Time to decannulation was also significantly shorter, by 5.4 days, in the care pathway group (p < 0.001). As seen in Table 2, patients in this group also had lower rates of nosocomial pneumonia, complication requiring return to ICU, and complication requiring return to OR for neck exploration, although these reductions did not reach statistical significance.

**Table 2:** Post-operative outcomes in patients undergoing head and neck oncologic resection with microvascular reconstruction

	**Control *(n = 62)***	**Pathway *(n = 56)***	**Reduction**	**p value**
**Mean length of stay (days)**	21.3	14.1	33.8%	< 0.001
**Return to OR**	16.1%	8.9%	44.7%	0.28
**Readmission to ICU**	12.9%	5.4%	59.5%	0.20
**Mean time to decannulation (days)**	13.6	8.2	39.7%	< 0.001
**Pneumonia,% (n)**	30.6%	17.9%	41.5%	0.10

The results of time-driven activity based costing are shown in Table 3. Costs are broken down into inpatient ward cost, share of return to ICU costs, and share of return to OR cost. Patients in the care pathway group had a mean total cost reduction of $6,169 (27.1%) per inpatient stay compared to the control cohort.

**Table 3:** Mean postoperative inpatient cost breakdown for patients undergoing head and neck oncologic resection with microvascular reconstruction

	**Control (CAD)**	**Pathway (CAD)**	**Incremental cost (CAD)**	**Cost reduction**
**Mean inpatient ward cost**	$15,975	$10,756	- $5219	32.7%
**Mean return to OR cost**	$883	$310	- $573	64.9%
**Mean ICU costs**	$5,875	$5,498	- $377	6.4%
**Mean total postoperative inpatient cost**	$22,733	$16,564	- $6,169	27.1%

The intervention examined in this study, the head and neck clinical care pathway improved all measured clinical outcomes and reduced cost, when compared to the traditional postoperative recovery experience. In terms of cost-effectiveness analysis (CEA), this is therefore considered a dominant clinical intervention, or quadrant II of the cost-effectiveness plane, and is considered cost-effective. In cases of dominant interventions, incremental cost-effectiveness ratios do not need to be determined, as there is no need to quantifiably measure whether outcomes outweigh cost, or vice versa.

## Discussion

Our group has prospectively designed a clinical care pathway that we have shown reduces the mean postoperative length of stay in this group of patients, as well as improving other clinical outcomes. We also observed decreased mean time to decannulation, which has been reported to improve patient swallowing outcomes [12]. CEA of these outcomes indicates that implementation of this care pathway both reduces cost and improves clinical outcomes compared to the traditional postoperative recovery experience.

When evaluating a new treatment method or care pattern involving a multi-disciplinary care team, it important to not only measure the effectiveness of the method in terms of clinical outcomes, but also to assess the cost of the treatment. CEA is a useful method to determine the most efficient way to allocate limited resources among different interventions and methods [13,14]. To our knowledge, this is the first time that a head and neck clinical care pathway has been examined for cost-effectiveness.

The surgical treatment of head and neck cancer is a significant economic burden on the healthcare system. The resource-intensive nature of the operative procedure itself, along with the often complicated and prolonged inpatient recovery contributes to this burden. The reported rate of complications during inpatient recovery is considerable, and often leads to longer hospital stays, increased cost, and increased mortality [5-9,15]. Postoperative pulmonary complications alone have been shown to increase mortality by 12.8% in these patients [8].

Clinical pathways may be an effective method of diminishing complications and reducing health care costs. Clinical pathways for a variety of diagnoses and procedures are quickly becoming standard of care at many institutions in the United States. This is likely driven by privatization and the underlying theme of providing quality care in the most cost-effective manner. This has not occurred to the same extent in Canada. To our knowledge, this is the first clinical care pathway for head and neck surgery with microvascular reconstruction implemented in Canada. As the cost of Canada’s health care system continues to rise out of proportion to its GDP, new treatment methods and protocols should be created based on cost-effectiveness evidence rather than theory or opinion [16,17].

There is further evidence showing that clinical care pathways for treating head and neck cancer maintain quality of care while decreasing expenses in the United States. Authors have reported savings ranging from $2,232 to $39,419 per patient [4,18-20]. Reducing a patient’s length of stay has been the most dominant factor in these cost savings. The breakdown of costs associated with treatment of HNSCC is dominated by hospitalization costs [3]. These studies have shown reduction of the total length of stay by 1.5-5 days and reduction of the average ICU stay by 0.5 days [4,18-20]. Although statistically negligible, these studies reported decreased postoperative pneumonia, malnutrition, and readmission rates, which could contribute to greater patient satisfaction [4]. Clinical care pathways employed in other medical specialties have also shown decreased ICU readmission rates [20].

The value of a clinical care pathway lies in ensuring that all interventions and tests are optimally scheduled, to reduce unnecessary delays. From our experience, we believe this translates to fewer complications and more efficient discharge planning. Having the pathway available to all involved healthcare professionals ensures that all are aware of the daily care and discharge planning goals for the patient. This facilitates communication between busy healthcare providers and reduces the need for confirming routine care through physicians, which can lead to delays. We feel these methodological improvements apply across Canadian centers.

When examining what has been previously published in the Canadian literature, outcomes in our patients treated prior to implementation of the pathway are comparable to those reported by other centers. Other authors have reported a mean length of stay of 22.9 days, time to decannulation of 10.0 days, rate of return to the operating room of 16.0%, and rate of pneumonia of 13.3% [12,21-23]. Additionally, mean total inpatient cost reported by Smeele et al. was comparable to our pre-pathway cost, at $23,600 per patient. As a result, we feel that our head and neck cancer experience *prior* to implementation of the care pathway was fairly generalizable and representative of other centers throughout the country. Clinical care pathways may be beneficial across these centers and provide cost-savings on a wider scale.

Beyond the operative and inpatient costs, post-discharge costs also contribute significantly to the economic burden of head and neck oncologic surgery. Previous studies have shown that 56.7% of patients have at least one emergency room visit or hospital admission in the two years following discharge. In their U.S. study, Amonkar et al. showed this post-discharge period translates to a mean total cost of $85 000 USD per patient [24]. Furthermore, Funk et al. have identified the mean total one-year cost of head and neck surgery with free flap reconstruction to be $150 000 USD per patient, which includes the post-discharge period [25]. We have shown the implementation of this clinical care pathway to be cost-effective in the inpatient period, however, cost-effectiveness in the post-discharge period has not been demonstrated in the literature. It would be prudent for future research to investigate whether these patients who are discharged from hospital in a more efficient timeline have increased healthcare utilization or complications after discharge. Our group intends to examine this question as part of a future study.

The potential limitations of this study include the inherent cost uncertainty involved in estimation models. While we feel that the time-driven activity-based costing model provides an acceptably low level of uncertainty, as supported by health economics literature, we plan on performing decision tree analysis with calculation of probabilistic sensitivity analysis in the future, to further ensure the accuracy of this costing data [26].

The pre-pathway group consisted of 62 patients treated over a four-year time period, while the pathway group included 56 patients over a one-year period. While this gives the impression of an increase in patient volumes, which could confound the results, this apparent increase is due to the fact that head and neck oncology was centralized to the study hospital in our city during this time period. Overall patient volumes across the city were similar between the two time periods. The members and level of experience of the clinical teams managing the patients was similar between the two groups.

Another potential limitation arises from the fact that the most responsible physicians (MRP’s) were unable to be blinded to a study with this design. While this is a potential source of bias, the care pathway largely involves influencing clinical decisions made by other health care providers (nursing, respiratory therapy, etc.) who were not explicitly informed of the ongoing study. The outcomes measurement aspect of this project was not “front and center” for any providers and we do not believe this biased the results. In addition, data collection was not done by the MRP’s, but by nursing staff who were unaware of the specifics or aims of this particular study.

## Conclusion

Surgical management of HNSCC represents a substantial economic burden on the health care system; therefore it is important to develop innovative measures to optimize the cost-effectiveness of our clinical interventions. This study has demonstrated that implementation of a postoperative care pathway in patients undergoing major head and neck oncologic surgery with microvascular reconstruction was cost-effective compared to those patients managed prior to implementing the clinical pathway.

## Competing interests

The authors declare that they have no competing interests.

## Authors’ contributions

JFD was involved in study design, data collection, analysis and writing of the manuscript. LR was involved in designing the cost analysis and editing the manuscript. JY was involved in data collection. TA was involved in data collection. SN was involved in statistical analysis and editing the manuscript. MH was involved in literature review and writing the manuscript. AL was involved in literature review. SC was involved in patient recruitment and editing. TM was involved in patient recruitment and editing. CS was involved in patient recruitment and editing. JCD was involved in creation of the pathway, data analysis, study design, and editing the manuscript. All authors read and approved the final manuscript.

## Supplementary Material

Additional file 1 University of Calgary Head and Neck Clinical Care Pathway. Click here for file

## References

[B1] Lang K Menzin J Earle CC Jacobson J Hsu M The economic cost of squamous cell cancer of the head and neck: findings from linked SEER-Medicare data Arch Otolaryngol Head Neck Surg 2004 42 11 1269 1275 10.1001/archotol.130.11.1269 15545580

[B2] Organization for Economic Cooperation and Devolopment Health Data 2011 - How Does Canada Compare 2012 OECD.org from: http://www.oecd.org/health/healthdata2011.htm

[B3] Chen AY Callender D Mansyur C Reyna K Limitone E The impact of clinical pathways on the practice of head and neck oncologic surgery Arch Otolaryngol Head Neck Surg 2000 42 332 326 10.1001/archotol.126.3.322 10722004

[B4] Hanna E Schultz S Doctor D Vural E Stern S Suen J Development and implementation of a clinical pathway for patients undergoing total laryngectomy: impact on cost and quality of care Arch Otolaryngol Head Neck Surg 1999 42 1247.1251 10555697 10.1001/archotol.125.11.1247

[B5] Jones NF Jarrahy R Song JI Kaufman MR Markowitz B Postoperative medical complications–not microsurgical complications–negatively influence the morbidity, mortality, and true costs after microsurgical reconstruction for head and neck cancer Plast Reconstr Surg 2007 42 2053 2060 10.1097/01.prs.0000260591.82762.b5 17519700

[B6] McCulloch TM Jensen NF Girod DA Tsue TT Weymuller EA Jr Risk factors for pulmonary complications in the postoperative head and neck surgery patient Head Neck 1997 42 372 377 10.1002/(SICI)1097-0347(199708)19:5<372::AID-HED2>3.0.CO;2-X 9243263

[B7] Rao MK Reilley TE Schuller DE Young DC Analysis of risk factors for postoperative pulmonary complications in head and neck surgery Laryngoscope 1992 42 45 47 1731156 10.1288/00005537-199201000-00008

[B8] Petrar S Bartlett C Hart RD MacDougall P Pulmonary complications after major head and neck surgery: a retrospective cohort study Laryngoscope 2012 42 1057 1061 10.1002/lary.23228 22447296

[B9] BuSaba NY Schaumberg DA Predictors of prolonged length of stay after major elective head and neck surgery Laryngoscope 2007 42 1756 1763 10.1097/MLG.0b013e3180de4d85 17690609

[B10] Kaplan R Anderson S Time-driven activity-based costing: a simpler and more powerful path to higher profits 2007 US: Harvard Business School Publishing Corporation 15559451

[B11] Kaplan RS Porter ME The big idea: How to solve the cost crisis in health care 2011 Harvard Business Review http://hbr.org/2011/09/how-to-solve-the-cost-crisis-in-health-care/ar/1 21939127

[B12] Brookes J Seikaly H Diamond C Mechor B Harris JR Prospective randomized trial comparing the effect of early suturing of tracheostomy sites on postoperative patient swallowing and rehabilitation J Otol 2006 42 2 77 82 10.2310/7070.2005.4035 16527024

[B13] Drummond MF O’Brien B Stoddart GL Torrance GW Methods for the Economic Evaluation of Health Care Programmes 1997 2 Oxford, New York, Toronto: xford Medical Publications, Oxford University Press 96 131

[B14] Kobelt G Health Economics: An Introduction to Economic Evaluation 2002 2 London, UK: Office of health economics 56 72

[B15] Tess BH Glenister M Rodrigues C Wagner Incidence of hospital-acquired infection and length of hospital stay Eur J Clin Micriobio Infect Dis Feb 1996 42 2 81 88 10.1007/BF01967579 8500486

[B16] Woolhandler S Campbell T Himmelstein DU Costs of health care administrationin the United States and Canada N Engl J Med 2003 42 768 775 10.1056/NEJMsa022033 12930930

[B17] Heacock D Brobst R A multidisciplinary approach to critical path development: a valuable CQI tool J Nurs Care Qual July 1994 42 38 41 7919442 10.1097/00001786-199407000-00007

[B18] Husbands JM Weber RS Karpati RL Clinical care pathways: decreasing resource utilization in head and neck surgical patients Otolaryngol Head Neck Surg 1999 42 755.759 10580233 10.1053/hn.1999.v121.a98217

[B19] Gendron M Lai S Weinstein G Chalian C Clinical care pathway of head and neck cancer. A valuable tool for decreasing resource utilization Arch Otolaryngol Head Neck Surg 2002 42 258 262 10.1001/archotol.128.3.258 11886340

[B20] Eichenberger A-S Haller G Cheseaux N Lechappe V Garnerin P Walder B A clinical pathway in a post-anaesthesia care unit to reduce length of stay, mortality and unplanned intensive care unit admission Eur J Anaesthesiol 2011 42 12 859 866 10.1097/EJA.0b013e328347dff5 21885983

[B21] Smeele LE Goldstein D Tsai V Gullane PJ Neligan P Brown D Irish JC Morbidity and cost differences between free flap reconstruction and pedicled flap reconstruction in oral and oropharyngeal cancer: matched control study J Otol 2006 Apr 42 2 102 107 10.2310/7070.2005.5001 16527028

[B22] Novakovic D Patel RS Goldstein DP Gullane PJ Salvage of failed free flaps used in head and neck reconstruction Head Neck Oncol 2009 Aug 21 42 33 10.1186/1758-3284-1-33 19698095 PMC2749848

[B23] Petrar S Bartlett C Hart RD MacDougall P Pulmonary complications after major head and neck surgery: a retrospective cohort study Laryngoscope 2012 May 42 5 1057 1061 10.1002/lary.23228 22447296

[B24] Amonkar MM Chastek B Samant N Teitelbaum A Economic burden of resected squamous cell carcinoma of the head and neck in a US managed-care population J Med Econ 2011 42 4 421 432 21619455 10.3111/13696998.2011.584096

[B25] Funk GF Karnell LH Whitehead S Paulino A Ricks J Smith RB Free tissue transfer versus pedicled flap cost in head and neck cancer Otolaryngol -- Head and Neck Surg 2002 42 205 212 10.1067/mhn.2002.127591 12297811

[B26] Barnett PG An improved set of standards for finding cost for cost-effectiveness analysis Med Care 2009 Jul 42 7 Suppl 1 S82 S88 19536018 10.1097/MLR.0b013e31819e1f3f

