# Supplementary material for: Cost-effectiveness analysis of a postoperative clinical care pathway in head and neck surgery with microvascular reconstruction
Source: J Otolaryngol Head Neck Surg. 2013 Dec 19;42(1):59. doi: 10.1186/1916-0216-42-59 (PMC3878235; doi:10.1186/1916-0216-42-59)
Supplement: Additional file 1 — University of Calgary Head and Neck Clinical Care Pathway. [file 1916-0216-42-59-S1.docx]

**Additional file 1. University of Calgary Head and Neck Clinical Care Pathway.**

|  | Day of Surgery (ICU) | POD-1 | POD-2 | POD-3 | POD-4 | POD-5 | POD-6 | POD-7 | POD-8 | POD-10 | Discharge Goals |
| --- | --- | --- | --- | --- | --- | --- | --- | --- | --- | --- | --- |
| **TRACH** | Sterile x 72 hours | Cuff deflated by RT |  | Routine trach care protocol |  | Change Trach tube to non-cuffed unless contraindicated | Start corking trials | Assess if ready for decannulation |  |  | Trach out or at home trach teaching completed |
| **FLAP DONOR SITE** | Donor remains dressed x 5 days |  |  |  |  | Dressing down – daily dressings start. OT to splint. |  |  |  | Dressing changed to glaxal if healed | Teaching or home care orders for daily dressing |
| **SKIN GRAFT DONOR SITE** |  | Remove outer covering over dressing |  |  |  | Staples removed |  |  |  |  | Site care education |
| **FLAP CHECKS** | Q1H x 48 hours |  | Q2H x 48 hours |  | Q4H x 48 hours |  | D/C flap checks |  |  |  |  |
| **DRAINS** |  |  |  |  |  | Discontinue drains |  |  |  |  |  |
| **LABORATORY** |  | CBC, lytes, Ca, Phos, Mg, Cr, Glu, INR | CBC, lytes, Ca, Phos, Mg, Cr, Glu, INR | CBC, lytes, Ca, Phos, Mg, Cr, Glu, INR |  | CBC, lytes, Ca, phos |  |  | CBC, lytes, Ca, Phos, Mg, Cr, Glu, INR |  |  |
| **VITAL SIGNS** | Q1H x 48 hours |  | Q2H x 48 hours |  | Q4H x 48 hours |  | Q8H |  |  |  |  |
| **SUTURE CARE** |  |  |  |  |  |  | Facial sutures removed |  | Neck sutures removed |  |  |
| **IV THERAPY/ANTIBIOTICS** | Antibiotics x 3 doses | TFI ordered |  | Adjust IV according to tube feed rate to TFI |  | IV saline locked |  |  |  |  |  |
| **PAIN MANAGEMENT** | PCA | PCA | PCA | PCA |  | D/C PCA – liquid meds via nasogastric |  |  |  |  | Oral/NG pain meds |
| **CIWA** | Start if appropriate |  |  |  |  |  |  |  |  |  |  |
| **ACTIVITY** | Bedrest |  | Sitting in bed or bedside | Activity as tolerated |  | Should be ambulating |  |  |  |  | Independently mobile or at baseline |
| **NUTRITION** | NPO to trickle feeds | Tube feed starts (continuous) |  |  |  | Start bolus feeds |  | Early g-tube PRN or discharge teaching for home feeds |  | FEES booked | Tolerating full fluid diet or tube feeds teaching completed |
| **ELIMINATION** |  |  |  | D/C Foley,  Bowel routine |  |  |  |  |  |  |  |
| **PHYSIOTHERAPY** |  | Chest physio ordered all patients |  |  |  |  | Physio to start full ROM | H&N PT prescription.  Prep for discharge. |  |  |  |
| **MISCELLANEOUS** | Oral rinses 15mL NS Q2H (use rubber catheter for suctioning) | PT, Dietician, social work, transition services consults | Cancer centre follow up re: appointments | Consult OT to splint on POD 5 |  | Switch NS rinses to chlorhexidine 0.12% - 25 mL BID x 7 days |  |  |  |  |  |

PCA: patient controlled analgesia OT: occupational therapy

NS: normal saline POD: post-operative day

PT: physiotherapy ROM: range of motion

RT: respiratory therapy FEES: functional endoscopic evaluation of swallowing
